# Nutrition in primary and secondary prevention of cardiovascular risk in the continental and Mediterranean regions of Croatia

**DOI:** 10.1186/s12872-017-0678-z

**Published:** 2017-09-16

**Authors:** Jozica Sikic, Mira Stipcevic, Hrvoje Vrazic, Jasna Cerkez Habek, Eduard Margetic, Dario Gulin

**Affiliations:** 1 Division of Cardiology, Department of Internal Medicine, Sveti Duh University Hospital, Zagreb, Croatia; 2 Division of Cardiology, Department of Internal Medicine, Dubrava University Hospital, Zagreb, Croatia; 3 University Clinic of Cardiovascular Diseases, Zagreb University Hospital Centre, Zagreb, Croatia; 4 School of Medicine, University of Zagreb, Zagreb, Croatia

**Keywords:** Coronary heart disease, Cardiovascular risk factor, Mediterranean diet, Croatia, Primary prevention, Secondary prevention

## Abstract

**Background:**

The aim of this observational study was to evaluate the effect of Mediterranean and continental nutrition on cardiovascular risk in patients with acute and chronic coronary heart disease in Croatia.

**Methods:**

The study included 1284 patients who were hospitalized in a 28-month period due to acute or chronic ischaemic heart disease in hospitals across Croatia. An individual questionnaire was prepared which enabled recording of various cardiovascular risk factors.

**Results:**

Patients with chronic coronary artery disease have a better index of healthy diet than patients with acute coronary disease. Women have a better index of diet than men in both Croatian regions. When the prevalence of risk factors (impaired glucose tolerance, diabetes mellitus types I and II, hypercholesterolaemia, hypertriglyceridaemia and hypertension) in patients with Mediterranean and continental nutrition is compared, a trend is seen for patients who have risk factors to consume healthier food.

**Conclusion:**

The Mediterranean diet is associated with reduced risk of developing cardiovascular disease. This effect is more evident in patients with known cardiovascular disease.

**Electronic supplementary material:**

The online version of this article (10.1186/s12872-017-0678-z) contains supplementary material, which is available to authorized users.

## Background

Cardiovascular diseases are the leading cause of death in industrialized countries, but their incidence has a significant geographical variation. The lower incidence of coronary heart disease (CHD) in the Mediterranean countries has been attributed mainly to dietary habits [1–3]. Previously published retrospective studies [4–6] have shown that adherence to a Mediterranean diet is associated with a significant reduction in mortality in continental countries, compared to Mediterranean countries [4, 5].

However, recent studies show a reduction in differences in the incidence of coronary heart disease, with regard to psychosocial and dietary habits, in the Mediterranean and continental countries [6, 7] due to education and greater availability of healthy food.

A study conducted in India showed a significant difference in the incidence of unstable angina between the inhabitants of the southern (coastal) and northern (continental) parts, with a higher incidence of hypertension and other risk factors in the north [8]. Begom et al. found that intake of diet high in saturated fat and cholesterol is higher in the inhabitants of the southern parts of India. The prevalence of CHD was 61.6% higher in South Indians (13.9 vs 8.6%) [9].

The Mediterranean diet is based on vegetables, olive oil and fish. Subjects who consumed olive oil had a lower mortality rate than those who consumed other types of fat [6, 7, 9–11]. According to several small clinical studies, the Mediterranean diet, or any of its components, has a significant impact on lowering blood pressure [12], reduction of serum cholesterol [13, 14] and improvement of endothelial function [15]. A cross-sectional study published in 2004 [16] and a two-year study of dietary habits [17] showed that such a diet, measured by various indices, is associated with reduced C-reactive protein (CRP), interleukin-6 and markers of endothelial function. Reductions in adipocytokine and adiponectin levels, which are associated with increased risk of cardiovascular disease (CVD), were also observed. According to Barbero et al., male sex, reduced ejection fraction, diabetes, prior MI, and high C-reactive protein were the most powerful predictors of cardiovascular events [18]. Meta-analysis from 2016 showed favourable effects of healthy dietary patterns on CRP, with limited evidence for other biomarkers [19–25].

A connection between the Mediterranean diet and reduction in level of oxidized low-density lipoprotein (LDL) and blood pressure was also observed [20]. This relationship is better than in patients on a hypolipaemic diet. The Mediterranean diet is clearly associated with a reduction in fatal and nonfatal CVD, which is probably a consequence of taking large quantities of fish in the diet [17–21]. These favourable effects on the biochemical level explain the epidemiological evidence of beneficial effects of a Mediterranean diet on the cardiovascular system. The aim of this study was to evaluate the effect of Mediterranean and continental food on cardiovascular risk in patients with acute and chronic coronary heart disease in Croatia.

## Methods

The survey included 1284 patients hospitalized between 1st October 2007 and 7th January 2010 for acute or chronic ischaemic heart disease in various hospitals in Croatia. Acute coronary disease was defined as unstable angina, myocardial infarction with or without ST elevation. Chronic coronary disease was defined as significant coronary stenosis (more than 70%), positive myocardial perfusion scintigraphy test, echo stress test or treadmill exercise stress test to ischaemia. It was performed in the above-mentioned period in Dubrava University Hospital (Zagreb), Sveti Duh University Hospital (Zagreb), Bjelovar General Hospital, Čakovec General Hospital, Karlovac General Hospital, Koprivnica General Hospital, Slavonski Brod General Hospital, Varaždin General Hospital, Rijeka University Hospital Centre, Pula General Hospital, Split University Hospital Centre, Dubrovnik General Hospital and Zadar General Hospital.

A special questionnaire was produced for this study which enabled the recording of the data required. The questionnaire was produced after a series of consultations with experts and the literature, and it was compiled on the model of large clinical trials conducted in Europe and Croatia [INTERHEART, EUROASPIRE (European action on secondary prevention by intervention to reduce events) I and II, EH-UH (Epidemiology of hypertension in Croatia), TASPIC-CRO (Treatment and secondary prevention of ischemic coronary events in Croatia)] (*Additional file 1). This allowed the investigators to be able to compare results efficiently. Most of the questions had multiple answers offered in advance to acquire greater accuracy.

Some of the data were obtained from the patient’s medical history (personal and family history): age, gender, information about ongoing treatment and discharge diagnosis. Type of food and method of preparation were tested by a standardized questionnaire. It included the frequency of consumption of specific nutrients on a daily, weekly and monthly basis as follows: red meat, cured meat products, fish, poultry, eggs, whole grains, refined grains, dairy products, fried and breaded food, soy-based products, salty food and snacks, salt, sweets, fruits, juices, nuts, beans, potatoes, green leafy vegetables, other vegetables (cooked or raw), pickled vegetables, sweetened beverages, olive oil, other vegetable oils, animal fats, and untreated filtered coffee. The questionnaire was designed in accordance with standards of epidemiological studies of chronic degenerative diseases. Dietary patterns were derived from factor analysis using food groupings. “Prudent pattern” and “Western pattern” (defined with prudent pattern score as diet index), as two types of dietary patterns were identified, predicting the incidence of CHD, independent of other lifestyle variables [26]. The reproducibility and validity of the food-frequency questionnaire (FFQ) used in this study was reported previously [27].

Data were collected by physicians or trained personnel (nurses), coded and entered into the electronic file. All patients signed a statement on consent to participate. Confidentiality of data was ensured in accordance with current applicable codes, declarations and other provisions. The results are shown in the tables, and for quantitative variables, descriptive statistics were produced with appropriate measures of central tendency and variability (mean, standard deviation, medians, associated interquartile ranges). Normal distribution of quantitative variables was tested by the Kolmogorov-Smirnov test, and then appropriate tests – parametric (*t*-test for independent samples and analysis of variance – ANOVA) or nonparametric (Mann-Whitney U test, Kruskal-Wallis test) – were made. The χ^2^ test was also used.

Statistically significant results were considered to be those with *p* values <0.05. Statistical analysis was made using the PASW software, version 17.02 (Chicago Inc., Il, http://www.spss.com).

## Results

**Fig. 1:**
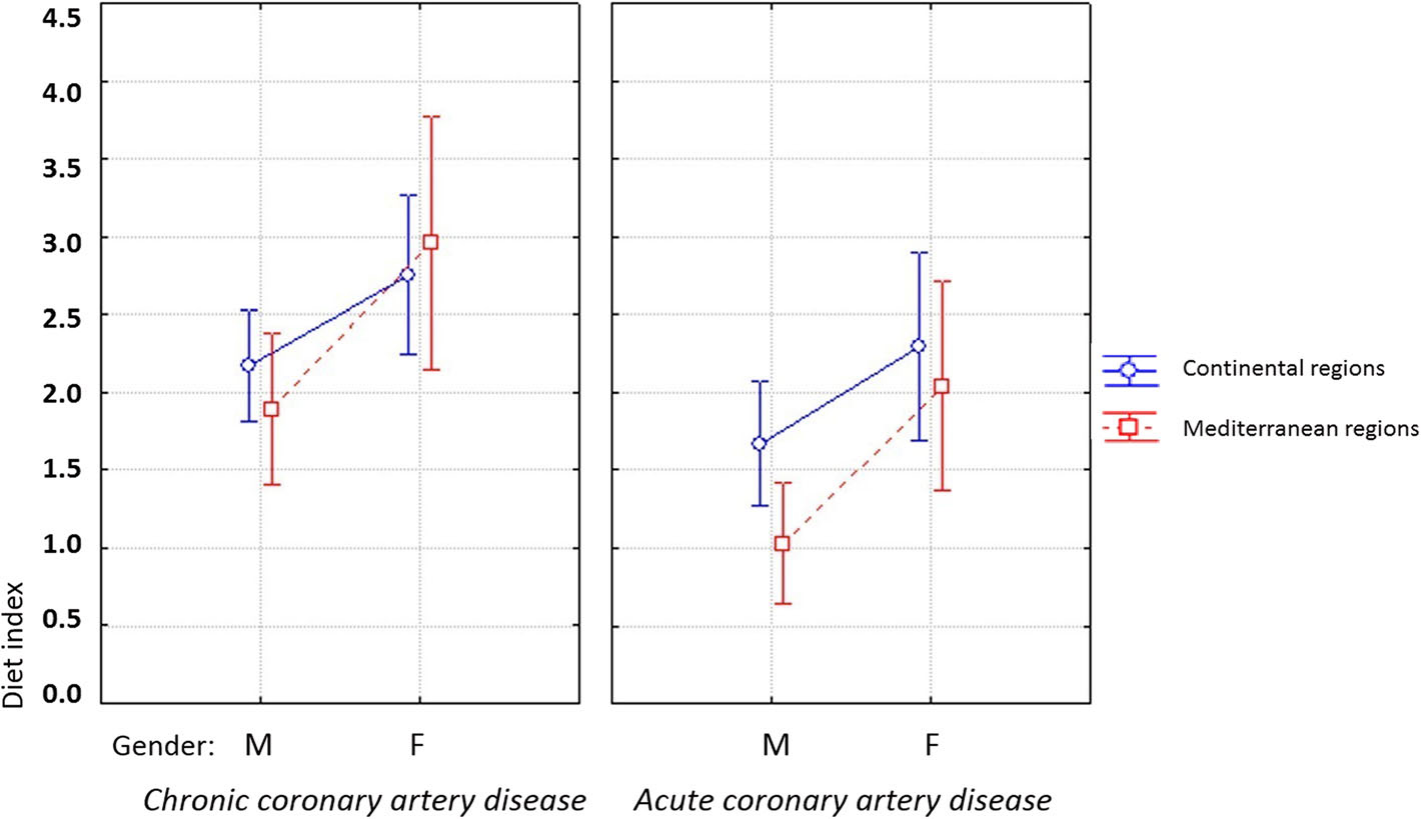
Type of diet (index) by gender, and acute/chronic coronary disease

This sub-analysis study included 1284 patients. Baseline characteristics of the patients studied are shown in Table 1. Figure 1 shows the distribution of subjects according to region, gender, and acute or chronic coronary disease against diet index. We noted that patients with chronic coronary heart disease eat more healthily than patients with acute coronary disease. A great variability in the mixture of Mediterranean and continental food is present in both regions, with high coefficients of variation (> 60%). In both regions, women and men equally consume continental food. A good diet factor is 1.8 for the continental region, and 1.6 for the Mediterranean. A poor diet factor is 1.4 for the continental region and 1.5 for the Mediterranean. No statistically significant differences in the quality of food between the regions were observed (Table 2).

**Table 1:** Baseline clinical characteristics of studied individuals

	Acute coronary artery disease (*n* = 655)	Chronic coronary artery disease (*n* = 629)	*p*
Age years Mean ± standard deviation	62.9 ± 12.1	63.2 ± 11.1	NS
Male age years Mean ± standard deviation	61 ± 11.6	61.4 ± 10.8	*p* < 0.0001
Female age years Mean ± standard deviation	67.8 ± 11.8	67.4 ± 10.7	
Female n (%)	181 (28)	191 (30)	NS
BMI male Mean ± standard deviation (kg/m^2^)	27.9 ± 4	28.3 ± 5	NS
BMI female Mean ± standard deviation (kg/m^2^)	28.5 ± 5.1	28 ± 4.4	NS
Obesity n (%)	367 (28.6)		
Diabetes n (%)	406 (31.6)		
Elevated cholesterol n (%)	924 (72)		
Hypertension n (%)	900 (70.1)		
Smoking	Non-smoker n (%)	444 (34.6)	
	Smoker n (%)	547 (42.6)	
	Ex-smoker n (%)	293 (22.8)

**Table 2:** Type of diet (index) by region, gender, and acute or chronic coronary disease

	N	Mean	SD	−95% CI	+95% CI
Total	1284	1,93	3,13	1,76	2,10
Continental region	751	2,14	3,11	1,92	2,36
Mediterranean region	533	1,63	3,14	1,36	1,89
Men	910	1,69	3,19	1,48	1,90
Women	374	2,51	2,90	2,21	2,80
Chronic coronary artery disease	629	2,30	3,13	2,06	2,55
Acute coronary artery disease	655	1,57	3,09	1,33	1,81

The frequency of fish consumption does not differ significantly between patients with acute coronary artery disease and those with a chronic condition. Eating meat products was significantly higher, by 100% (OR 2.00), skimmed milk by 46% (OR 1.46) and whole-fat milk by 45% (OR 1.45), in patients hospitalized for acute coronary heart disease. Consuming olive oil is also more common in patients hospitalized for acute coronary heart disease, by 68% (OR = 1.68, 95% CI 1.18 to 2.38, *p* = 0.0038). Patients hospitalized for acute coronary heart disease were significantly less likely to take statin therapy (OR = 0.46, 95% CI 0.35 to 0.60; *p* < 0.001) than patients hospitalized for chronic coronary heart disease (Table 3).

**Table 3:** Odds Ratio for occurrence of acute compared to chronic coronary disease with regard to certain food products

Type of food	OR	CI 95%	p
Red meat	1,84	0,99–3,45	p = 0,0547
Cured meat	2,00	1,29–3,11	***p = 0,0020***
Poultry	0,33	0,15–0,73	***p = 0,0062***
Skimmed milk	1,46	1,04–2,05	***p= 0,0302***
Whole milk	1,45	1,05–2,00	***p = 0,0254***
Fruit	0,56	0,33–0,95	***p = 0,0321***
Fish	1,00	0,68–1,49	P = 0,9836
Olive oil	1,68	1,18–2,38	***p = 0,0038***
Not taking statins	0,46	0,35–0,60	***p < 0,001***

Bolded and italics present statistically significant values

Men and women with chronic coronary artery disease eat healthier food than patients with acute coronary disease in both regions. Patients hospitalized for chronic coronary artery disease had a better index of healthy diet than patients with acute coronary disease (2.30 vs 1.57; *p* = 0.0006). Women had a better index of diet than men in both regions (2.51 vs 1.69; *p* < 0.0001). Diet index in the women’s group was 2.56 in the continental region and 2.41 in the Mediterranean. In the men’s group it was 1.95 in the continental region and 1.36 in the Mediterranean.

Men with acute coronary disease in the continental region consumed healthier food than men in the Mediterranean region (diet index 1.86 vs. 1.28; *p* = 0.0391). In men with chronic coronary artery disease, there were no significant differences in the quality of food given to the region. In women with acute coronary disease, there were no statistically significant differences in type of food with regard to region. Women with chronic coronary artery disease consumed mixed nutrients in both regions. The presence of a healthy diet was higher in women with chronic coronary artery disease of the Mediterranean region (diet index 2.96 vs. 2.76; *p* = 0.0485).

With respect to the prevalence of risk factors (elevated blood glucose, a type I DM, hyperlipidaemia, hypertriglyceridaemia, hypertension) in patients consuming Mediterranean and continental food, analysis of variance did not yield a statistically significant difference in the incidence of the same, although there was a trend for patients who had risk factors to eat healthier (*p* < 0.1). For patients with diabetes mellitus (DM) type II, it was statistically significant (*p* = 0.00004).

## Discussion

In this study, no differences were found in diet by region; however, patients who had known cardiovascular disease tended to have a healthier diet than those who did not. Although this study has limitations, it brings valuable data about healthier eating patterns in patients with chronic heart disease. It is an observational study, and it could be affected by self-reporting bias in which FFQs are very unreliable, where patients are more likely to answer in a way to please the researchers making them look as good as possible. Also, it could be impacted by the reporting not only short-term intake but also long-term dietary exposure without measuring actual food intake. Patients with chronic heart disease are mainly familiar with the type of diet they are supposed to consume. Following that, it is not uncommon that certain types of food are underreported and others, such as olive oil, fruit, and vegetable, reported more, trying to minimize inability to implement healthy eating patterns.

Compared to those with chronic disease, those who presented with acute coronary syndrome were more like to eat cured and red meat, dairy products and olive oil and less likely to eat fruit and vegetables (Fig. 1; Table 3). This can be interpreted in terms of better education of patients with chronic coronary disease. According to a study of 7 European countries, members of higher socioeconomic classes consume healthier food [28–30].

According to our study, fish consumption was not significantly different in patients with acute or chronic coronary disease. This is probably because of a generally low level of fish consumption in Croatia, which clearly does not change with education or presence of risk factors for cardiovascular disease. (Citizens of Croatia, per capita per year, consume around 10 kg of fish, while this is 21 kg in Greece, 24 kg in Italy, 33 kg in France, 44 kg in Spain, 57 kg in Portugal and, in Iceland, an elusive 90 kg of fish). A meta-analysis showed that eating fish less than once a month increases the risk of developing coronary heart disease in comparison to subjects who consume fish once a week, two to four times a week, or more than four times a week (0.89 by 0.85 by 0.77 to 0.62) [31].

Hospitalizations for acute, rather than chronic, coronary artery disease were associated, in our patients, with an increased odds ratio for consumption of olive oil by 68%. In fact, due to their greater dedication to education, patients with other risk factors, such as hypertension, hyperlipidaemia and diabetes mellitus, try to eat more healthily, so in our case a healthy diet, in connection with certain nutrients, reflects risk more than actual prevention. It is thought that some dietary habits could replace adequate cardiac rehabilitation and life-style modification. That kind of ‘myth’ is considered to hold for olive oil in Croatia, probably based on its being considered sufficient to introduce olive oil into the diet, with no other interventions in nutrition or other lifestyle habits, to significantly reduce risk and prevent the development of coronary disease [32–34].

Consuming low-fat meat, fish and other seafood is more common in people of higher socioeconomic status, while members of lower socioeconomic status more frequently consume food fried in oil and fat, with less protective fibre, less natural vitamin C, vitamin D, β-carotene, folate and vitamin E [28–30]. It seems that fibrin fibres (originating mostly from grain, then from fruit and vegetables) have a protective effect on the development of coronary heart disease and obesity, among other things, by reducing the level of insulin in the blood [35, 36].

## Conclusions

In Croatia, those with known chronic heart disease have a healthier eating pattern than those who present for the first time with acute coronary syndrome, regardless of historical geographic differences in eating patterns.

## Additional file

Additional file 1 *Questionnaire English version, *Study questionnaire containing main personal information, laboratory and physical findings and diet habits. (PDF 316 kb)

## Abbreviations

CHD: coronary heart disease
CRP: C-reactive protein
CVD: cardiovascular disease
DM: diabetes mellitus
LDL: low-density lipoprotein
