# Supplementary material for: Nutrition in primary and secondary prevention of cardiovascular risk in the continental and Mediterranean regions of Croatia
Source: BMC Cardiovasc Disord. 2017 Sep 16;17:247. doi: 10.1186/s12872-017-0678-z (PMC5603055; doi:10.1186/s12872-017-0678-z)
Supplement: Additional file 1 — *Questionnaire English version, *Study questionnaire containing main personal information, laboratory and physical findings and diet habits. (PDF 316 kb) [file 12872_2017_678_MOESM1_ESM.pdf]

Clinical Study	Institution	Family name	Personal name	Patient´s initials

Personal information

Sex:
- □ male
- □ female

Study inclusion - Date and time signing the Informed Consent Form

□□□□□□□□ □□:□□

YEAR (YYYY) MONTH (MM) DAY (DD) HOUR (HH) MINUTE (MM)

Name: □□□□□□□□□□□□□□□□□□

□□□□□□□□□□□□□□□□□□

Surname: □□□□□□□□□□□□□□□□□□

□□□□□□□□□□□□□□□□□□

Date of birth: □□ □□□ □□□□

Home address: □□□□□□□□□□□□□□□□□□□

Postal code and city: □□□□□□□□□□□□□□□□□□□

Tel. number: □□□□□□□□□□□□□□□□□

Phone number: □□□□□□□□□□□□□□□□□

Laboratory findings:

Date and time:<br>□□ □□□ □□□□ □□:□□	Fasting:<br>□ yes<br>□ no

Lab value:	
Glucose: □□.□□ mmol/L	CRP: □□.□□ mg/L
Cholesterol:<br>□□□□. mmol/L	Triglycerides:<br>□□□□. mmol/L
HDL:<br>□□.□□ mmol/L	LDL:<br>□□.□□ mmol/L

Physical findings:

Date and time:<br>□□ □□□ □□□□ □□:□□

Weight: □□□ **kg** | **Height:** □□□ **cm**

Waist circumference: □□□.□cm **Hip circumference**: □□□.□ cm

Blood pressure: **Pulse:** □□□**beats/min**

First measurement: **Left hand:** □□□/□□□ **mmHg** **Right hand:** □□□/□□□ **mmHg**

Second measurement: **Left hand:** □□□/□□□ **mmHg** **Right hand:** □□□/□□□ **mmHg**

Diet habits:

Are you a vegetarian? ◻ **YES** ◻ **NO**

How often do you eat food from these categories?

	Choose one answer				
	< 1x monthly or never	monthly	weekly	daily	If chosen monthly or weakly, how often
Red meat	◻	◻	◻	◻	
Cured meat products	◻	◻	◻	◻	
Poultry	◻	◻	◻	◻	
Fish	◻	◻	◻	◻	
Eggs	◻	◻	◻	◻	
Whole grains	◻	◻	◻	◻	
Rafined grains	◻	◻	◻	◻	
Dairy products	◻	◻	◻	◻	
Fried and breaded food	◻	◻	◻	◻	
Soy-based food	◻	◻	◻	◻	
Salty food and snacks	◻	◻	◻	◻	
Sweets	◻	◻	◻	◻	
Salt	◻	◻	◻	◻	
Fruits	◻	◻	◻	◻	
Juices	◻	◻	◻	◻	
Nuts	◻	◻	◻	◻	
Beans	◻	◻	◻	◻	
Potatoes	◻	◻	◻	◻	
Green leafy vegetables	◻	◻	◻	◻	
Pickled vegetables	◻	◻	◻	◻	
Other vegetables (cooked or raw)	◻	◻	◻	◻	
Sweetened beverages	◻	◻	◻	◻	
Olive oil	◻	◻	◻	◻	
Other vegetable oils	◻	◻	◻	◻	
Animal fats	◻	◻	◻	◻	
Untreated filtered coffee	◻	◻	◻	◻	

Smoking habits:

Have you ever used any of these tobacco products?

	Never	Before	Duration	Now	No per day
Cigarettes	◻	◻		◻	

**Ciagrs/tobacco pipe**	◻	◻		◻	
**Chewing tobacco**	◻	◻		◻	
**Other:___________________________**					

36.
